# Minimizing Fuel Consumption for Surveillance Unmanned Aerial Vehicles Using Parallel Particle Swarm Optimization

**DOI:** 10.3390/s24020408

**Published:** 2024-01-09

**Authors:** Vincent Roberge, Gilles Labonté, Mohammed Tarbouchi

**Affiliations:** 1 Department of Electrical and Computer Engineering, Royal Military College of Canada, Kingston, ON K7K 7B4, Canada; tarbouchi-m@rmc.ca; 2 Department of Mathematics and Computer Science, Royal Military College of Canada, Kingston, ON K7K 7B4, Canada; labonte-g@rmc.ca

**Keywords:** unmanned aerial vehicle, surveillance, particle swarm optimization, fuel consumption, equation of motion, optimization

## Abstract

This paper presents a method based on particle swarm optimization (PSO) for optimizing the power settings of unmanned aerial vehicle (UAVs) along a given trajectory in order to minimize fuel consumption and maximize autonomy during surveillance missions. UAVs are widely used in surveillance missions and their autonomy is a key characteristic that contributes to their success. Providing a way to reduce fuel consumption and increase autonomy provides a significant advantage during the mission. The method proposed in this paper included path smoothing techniques in 3D for fixed-wing UAVs based on circular arcs that overfly the waypoints, an essential feature in a surveillance mission. It used the equations of motions and the decomposition of Newton’s equation to compute the fuel consumption based on a given power setting. The proposed method used PSO to compute optimized power settings while respecting the absolute physical constraints, such as the load factor, the lift coefficient, the maximum speed and the maximum amount of fuel onboard. Finally, the method was parallelized on a multicore processor to accelerate the computation and provide fast optimization of the power settings in case the trajectory was changed in flight by the operator. Our results showed that the proposed PSO was able to reduce fuel consumption by up to 25% in the trajectories tested and the parallel implementation provided a speedup of 21.67× compared to a sequential implementation on the CPU.

## 1. Introduction

This paper deals with the problem of minimizing fuel consumption for fixed-wing propeller UAVs. This is important in a surveillance mission as reducing the fuel consumption of the UAV increases its autonomy and its range, allowing for longer distances to be patrolled and longer times to be spent on targets. However, minimizing fuel consumption for aircraft is difficult due to the complexity of estimating the fuel consumption. In fact, most of the methods previously used to estimate the fuel consumption of aircraft have relied on precomputed or experimentally recorded tables [1]. This may be sufficient for commercial aircraft, which typically fly predetermined trajectories, but cannot be used efficiently for UAVs, which fly ever-changing paths based on the progressing circumstances of the mission. For the case of UAVs, it would be advantageous to have an accurate mathematical model to compute the fuel burn. Having such a model could also mean that it would be possible to develop optimization algorithms for minimizing fuel consumption along the trajectory.

Previous works that have focused on the estimation of fuel consumption for aircraft include [1], which used a machine learning method, namely the support vector method, to train a model from fuel consumption tables in order to estimate the consumption for an unseen trajectory. In [2], Xi and Jingjie used a similar support vector-based approach but improved it with concepts from the just-in-time learning algorithm to focus the selection of the relevant sampling set and concepts from the differential evolution and tune the parameters of the support vector machine algorithm. Using their enhanced approach, they achieved superior estimation results. Another example based on pre-calculated tables was proposed in [3] where Zhang et al. used linear regression to estimate the fuel consumption of a flight path. One other example using pre-calculated or experimental data was from [4], where the fuel consumption was estimated using the RELAX algorithm. This algorithm relies on a dataset and uses a very large number of repetitive iterations of signal components to approximate signal parameters for the estimation of fuel consumption.

Other methods have relied on developing analytical models to directly calculate the fuel consumption instead of relying on experimental datasets. This is the case of [5], in which L’Afflitto and Sultan modeled the aircraft as a six degrees-of-freedom rigid body. However, their method made several assumptions and did not consider the altitude of the aircraft or the propeller efficiency. Another work relying on an analytical model was proposed by Wang et al. in [6] for propulsion aircraft and can, therefore, not be used for propeller aircraft, which is often the case for UAVs. One of the most complete works using an analytical model to compute the fuel consumption of propeller aircraft was published by us in 2012 [7]. The approach used Newton’s second law of motion to derive the equations involved. These were Riccati equations or were reduced to such equations after neglecting a small term. The equations were then transformed into second order linear differential equations that were solved exactly. Despite the rigor of this work, there were still approximations and the equations were valid for flight at constant speeds only. To minimize fuel consumption along a trajectory by varying the power setting and velocity of the UAV, it would be important to have equations for fuel consumption that are based on the power settings of the UAV and not its speed.

Due to the lack of accurate methods for computing fuel consumption, very few methods exist to minimize fuel consumption along a trajectory. In [8], Frazzoli et al. used a simulated annealing metaheuristic algorithm supplemented with a Monte Carlo simulation to minimize the aircraft fuel consumption. Their model for the fuel burn was not based on analytical formulas, but on a point mass model with the aircraft performance parameters derived from an online database. One last example of fuel minimization was provided in [9], where Brown and Anderson used PSO to compute a trajectory for a maritime radar surveillance UAV that minimized fuel consumption. The fitness function relies on a simplified analytical model to compute the fuel consumption associated with a trajectory. In most cases, these simplified models compute the fuel consumption based on a given aircraft velocity. In fact, we show in this work that this cannot be done accurately, and that one can only compute the velocity and fuel consumption of a UAV based on a given power setting.

**Figure 1:**
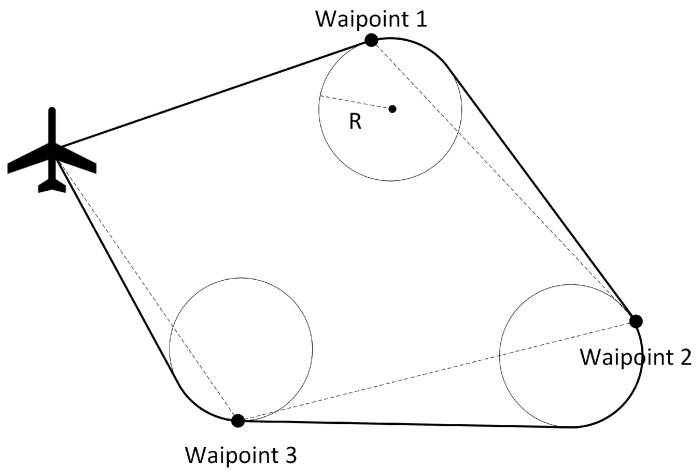
Example of a trajectory overflying the waypoints smoothed with circular arcs.

This paper presents a method based on PSO for optimizing the power settings of a UAV along a given trajectory in order to minimize fuel consumption and maximize autonomy. Given a trajectory represented by a series of waypoints, such as the one shown in Figure 1, our proposed approach first used a method inspired from [10] to smooth the trajectory using circular arcs. However, what is unique to our work is that the geometrical constructions were arranged so that the smoothed trajectory overflew the waypoint, which is desired in a surveillance mission or for UAVs tasked with collecting data from distributed wireless sensors, such those as described in [11]. Secondly, our method segmented the trajectory into a large number of small segments and computed the power settings for each segment using PSO.

One downside of using PSO or most metaheuristics in general is that their computation time can be long as they work by iteratively improving candidate solutions over several iterations. To address this drawback, we resorted to parallel programming and implemented PSO in parallel on a multicore CPU. This solution has been used in the literature and three main approaches exist for this. The first one is to parallelize the evaluation of the fitness function [12]. This approach is known as the primary–secondary architecture, where the primary thread delegates the heavy computation to the secondary threads. Another approach is the island model, where each thread runs an independent instance of PSO and they exchange their solutions based on a given policy. This approach is more complicated to implement but has the advantage of limiting communication between the threads and maximizing the fraction of the code that is parallelized. This approach is discussed in detailed in [13]. The last approach is data-level parallelization [12], where low-level computations are parallelized based on partitioning the data on multiple cores. This method is mostly used in graphics processing units (GPUs) [14], but can also be used in CPUs. In this work, we used a mix of the primary–secondary method and data-level parallelization in that the fitness function of PSO was parallelized using multiple threads and the loops of PSO were parallelized using a data-level approach. This provided the greatest acceleration.

The contribution of this paper is fourfold. First, it provides a geometrical model for smoothing a trajectory in 3D while overflying the waypoints, which is essential in surveillance missions. Second, it provides an analytical formulation of the equation of motion for an aircraft to accurately compute the velocity and fuel consumption based on a given power setting. Third, it presents PSO that computes the power settings along the trajectory that minimize fuel consumption and maximize autonomy. Fourth, it parallelizes the proposed method in a multicore CPU, achieving a 21.67× speedup compared to a sequential execution. As demonstrated in the results section, this allowed for a 56.3 km trajectory to be optimized in just 22.36 s.

The analytical model used was the one found in classical textbooks, such as [15] and [16]. However, we incorporated a term that corresponds to the change in mass due to fuel consumption. This has been rarely done, but it is important as fuel consumption reduces with the weight of the aircraft. We then decomposed the equations in the Frenet–Serret coordinates and arranged them so that they could be resolved using a Runge–Kutta method for a given power setting.

**Figure 2:**
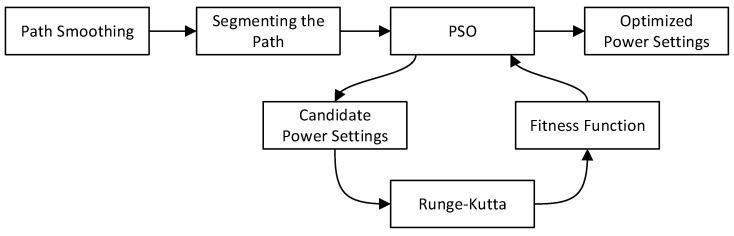
Overall process of the proposed PSO for fuel consumption minimization.

The relationships between the modules presented in this paper are illustrated in Figure 2. Given a trajectory as a series of waypoints, the initial step was to smooth the trajectory by connecting the line segments using circular arcs in a 3D space. Once the trajectory was smoothed, the long segments were divided into equal parts. This ensured that a power setting could be computed for each smaller segment. Then, PSO processed the overall trajectory in batches of 20 segments with an overlap of 10 segments between the sections. During the execution of PSO, each candidate solution (i.e., a sequence of 20 power settings) was tested using the Runge–Kutta method and the analytical model for fuel consumption. Once the fuel consumption and speed of the UAV was computed along the trajectory, the fitness function could be evaluated, which included the various physical constraints related to the UAV. Finally, the optimized power settings were returned by the program.

The remainder of this paper is organized as follows. Section 2 describes the method used to smooth the trajectory using circular arcs so that it is flyable by a fixed-wing UAV while still overflying the waypoints. Section 3 presents the equations of motion for the UAV with the decomposition of Newton’s equation, the absolute physical constraints related to the UAV and the trajectories flown at a prescribed power. Section 4 introduces PSO and explains how it was used in this research to compute the optimized power settings for the UAV along the trajectory. Finally, Section 5 presents the results with a focus on the quality of the solution computed and the acceleration provided by the parallel implementation in a multicore CPU for fast computation.

## 2. Trajectory Smoothing Using Circular Arcs

A much-used and efficient method for generating paths for fixed-wing UAVs consists of providing points from which a stickman path can be constructed as a continuous chain of rectilinear segments linked one to another. This path is then smoothed out by replacing the sharp corners where the rectilinear segments meet by continuous curves. This should ensure the continuity of the path tangent, as this continuity corresponds to the continuity of the velocity of the airplane. Since the dynamics of airplanes on circular arcs are relatively easy to analyze, this is the type of connection that is preferred in path construction, and the one that we considered here.

The points provided can play two different roles. In the first one, they end up being outside of the path and a bypass arc of a circle is introduced inside the acute angle made by the rectilinear segments. This arc of a circle is tangent to the two rectilinear segments. Such points can be termed “*control points*” as their role consists of shaping the global path. Alternatively, the path could go through the provided points, which then become true “*waypoints*”. In this research, we examined this second approach in which the path went through the waypoint.

**Figure 3:**
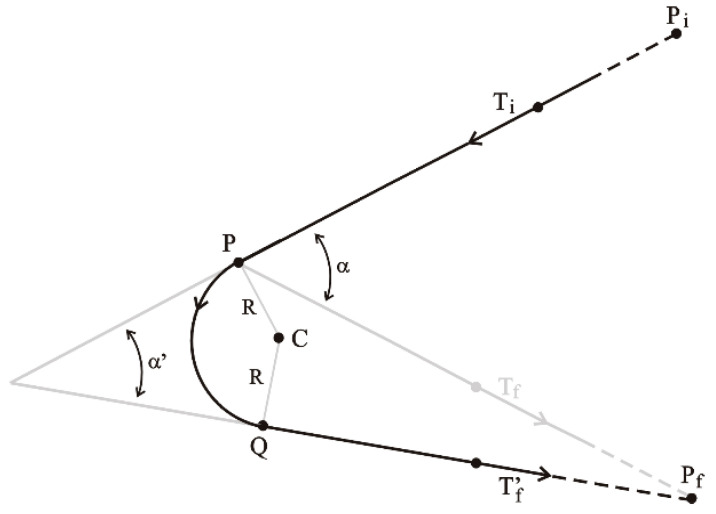
A circular connection from the incoming segment ***P****_i_**P*** to the outgoing segment ***PP****_f_* that goes through point ***P***.

We considered a passage from the incoming segment ***P****_i_**P*** to the outgoing segment ***PP****_f_* that went through point ***P***. Figure 3 shows such a connection as seen directly from above the plane in which it lies, together with some geometrical constructions that we used to describe it.

Let ***T_i_*** and ***T_f_*** denote the respective unit tangent vectors for the two rectilinear segments ***P_i_P*** and ***PP_f_***. The angle *α* between these segments is as follows.

***T****_i_·**T**_f_* = −cos(*α*). (1)

Let $T_{i}^{⊥}$ denote the unit vector that is orthogonal to ***T_i_*** and points toward the inside of the angle ***P****_i_ **P P**_f_*. Then, the center ***C*** of the connecting circular arc is as follows.

(2) $$C=P+\;RT_{i}^{⊥}.$$

Let $T_{f}^{’}$ denote the unit tangent vector for the rectilinear segment ***CP**_f_* and *α’*. Then, the angle between ***T_i_*** and −$T_{f}^{’}$ is as follows.

(3) $$T_{i}\cdot T_{f}^{’}=-\cos(\alpha^{’})$$

An equation describing the circular arc can be obtained by defining the two orthogonal unit vectors ***p*** and ***q*** as follows.

(4) $$p=\frac{1}{2\cos\left(a’/2\right)}\left[T_{i}-T_{f}^{’}\right]$$

(5) $$\mathrm{and}\;\;\;\;q=\frac{1}{2\sin\left(a’/2\right)}\left[T_{i}+T_{f}^{’}\right].$$

so that the points ***x***(*ϕ*) on the circular arc are as follows.

(6) $$\begin{array}{cc} & x(\phi )\;=\;C\;+\;R\;[p\;\cos(\phi )\;+\;q\;\sin(\phi )]\\ \mathrm{with} & \phi \in \;[-(\pi -a^{’})/2,\;(\pi -\;a^{’})/2]. \end{array}$$

where the angle *ϕ* is null at the midpoint between ***P*** and ***Q*** and increases in the counter-clockwise direction around the center ***C*** of the circle, in the plane of the path, from ***P*** at *ϕ* = −(*π* − α’ )/2 to ***Q*** at *ϕ* = (*π* – α’ )/2.

## 3. Equations of Motion for UAVs

The motion of airplanes is regulated by the power generated by its engines. In [15], it was explained that, because of their internal combustion nature, engines produce power that varies with altitude as the air density changes, according to the following equation.

(7) $$P\left(h\right)=P(0)\frac{\rho_{\infty }\left(h\right)}{r_{s}}$$

in which *ρ_∞_*(h) is the density of the undisturbed air in front of the airplane at altitude *h* and *ρ_s_* and *P*(*0*) are, respectively, the values of *ρ_∞_* and *P* at sea level. Most of the power produced by the engines is transferred to the propellers that receive the power *P_A_*, as shown in Equation (8).

*P_A_ = ηP* (8)

The parameter *η* is the efficiency of the propeller, which varies with the speed of the airplane. The available power *P_A_* must be at least equal to the power required *P_R_* for the airplane motion, which is determined by the equations of motion. The rate of fuel burning is described by the following equation.

(9) $$\frac{dW}{dt}=-cP$$

in which *W* is the total weight of the airplane and *c* is the specific fuel consumption. Chapter 5 of [16] explains how thrust is related to power for propeller driven airplanes. The propeller power *P_A_* moves the air with thrust *T_A_*, as shown in Equation (10).

*P_A_ = T_A_* (*V_∞_* + Δ*V_i_*). (10)

where *V_∞_* is the airplane speed and Δ*V_i_* is the speed increase of the air across the propeller disk. Even when the airplane is not moving, there is power required to turn the propellers. The available thrust *T_A_* is related to the power *P_A_* through a cubic equation, the solution of which is as follows.

(11) $$T_{A}(h,V_{\infty },P_{A})=P_{A}^{1/2}{\left(r_{\infty }A\right)}^{1/3}\left\{{\left[P_{A}^{1/2}-\sqrt{P_{A}+\frac{8r_{\infty }AV_{\infty }^{3}}{27}}\right]}^{1/3}+{\left[P_{A}^{1/2}+\sqrt{P_{A}+\frac{8r_{\infty }AV_{\infty }^{3}}{27}}\right]}^{1/3}\right\}$$

where *A = π Rad*^2^ is the area traced by the propeller of radius *Rad* when it rotates.

In [7], it was shown that if Newton’s equation of motion takes into account the change in the mass *M* of the airplane as fuel is burned, it takes the form of the following equation.

(12) $$M\frac{dv}{dt}-\left(AFR\right)\left[\;\frac{dM\;}{dt}\right]v=F.$$

In this equation, ***v*** is the airplane velocity, (AFR) is the air to fuel ratio in the combustion process, which is about 14.7 for gasoline or diesel [17], and ***F*** is the total force acting on the center of mass of the airplane. ***F*** has four components: the thrust ***T****_R_* produced by the engines, the lift produced by the airfoil and the airplane body, the drag due to air resistance and the force of gravity. The unit vector ***T*** is defined as being in the direction of the motion of the center of mass of the airplane. It is, therefore, tangent to the path and we considered that the thrust acts along its direction, as shown in Equation (13).

(13) $$T_{R}=T_{R\;}T,$$

The lift ***L*** is shown as follows.

(14) $$L={LU}_{L}\;\mathrm{with}\;L=\frac{1}{2}{\rho_{\infty }S\;C}_{L}V_{\infty }^{2},$$

where ***U****_L_* is a unit vector. Assuming that the airplane is bilaterally symmetric, we let ***w*** be the unit vector along the straight line from its left to its right wing tips. Then, ***U****_L_* = ***w*** *×* ***T*** so that it is always perpendicular to the direction of motion. The drag is shown as follows.

(15) $$D=-DT\mathrm{with}\;D=\frac{1}{2}{r_{\infty }S\;C}_{D}V_{\infty }^{2}$$

and the force of gravity is shown as follows.

(16) W=−Mgk,

in which *g* is the gravitational constant and ***k*** is the unit vector in the positive direction of the Earth *z*-axis.

### 3.1. Decomposition of Newton’s Equation

With the values of the force ***F*** given in Equations (13) to (16), Newton’s Equation (12) becomes the following.

(17) $$M\left[\frac{dV_{\infty }}{dt}T+\frac{V_{\infty }^{2}}{R}N\right]-\left(AFR\right)\frac{dM}{dt}V_{\infty }T=T_{R}T+LU_{L}-DT-Mgk$$

where ***T*** and ***N*** are, respectively, the Frenet–Serret unit tangent and unit normal vectors. The projection of Equation (17) along the vector ***T*** yields the following equation for the longitudinal motion.

(18) $$M\frac{dV_{\infty }}{dt}-\left(AFR\right)\frac{dM}{dt}V_{\infty }=T_{R}-D-Mg(k\cdot T).$$

There are two components of Equation (17) that are perpendicular to ***T***: one in the direction of the normal ***N*** and one in the direction of the binormal ***B***. Its component along ***N*** is shown in Equation (19).

(19) $$L\;{(U}_{L}\cdot N)=WA_{c}\;\;\mathrm{in}\;\mathrm{which}\;\;A_{c}=\frac{{\kappa \;V}_{\infty }^{2}}{g\;}+(k\cdot N)$$

is the centripetal acceleration. Projecting Equation (17) in the direction of ***B*** results in Equation (20).

(20) $$L{(U}_{L}\cdot \;B)=W(k\cdot B)\;.$$

Given Equations (19) and (20) and the fact that ***U****_L_* only has components along ***N*** and ***B***, it can be written as Equation (21).

(21) $$U_{L}=\frac{W}{L}\left[A_{c}N+(k\cdot B)B\right]\;.$$

Thus,

*L* = *Wn*, (22)

with

(23) $$n=\sqrt{A_{c}^{2}+{\left(k\cdot B\right)}^{2}}$$

### 3.2. The Absolute Physical Constraints

All airplanes are subject to constraints that are due to their construction and the power of their engines. These are as follows.

- The load factor *n* is bounded below by *n_min_* and above by *n_max_*, with *n_max_ >* 1 and *n_min_ ≤* −1.
- The lift coefficient is bounded below by *C_Lmin_* and above by *C_Lmax_*.
- The speed *V_∞_* is bounded below by the stall speed *V_stall_* at which the lift is not sufficient to sustain the airplane motion. It is bounded above by the value *V_NE_* (the suffix NE stands for “never exceed”), which is determined by the airplane construction. The power available to move the airplane is bounded above according to the capacity of its engines.
- There is also obviously a constraint on the fuel that is available.

For curved paths, the constraint on the load factor and on the lift coefficient impose a lower limit on the turning radius of the path.

### 3.3. Trajectories with Prescribed Power

We considered the situation in which the power provided by the engine is specified along the path as a continuous function of the distance *s* travelled along the path, as *P*(s) for *s* = *0* to *s_f_* = the length of the path.

There were three differential equations to solve. The first one was Equation (2), in which *P = P*(*s*). The second one was Equation (18), which described the longitudinal component of Newton’s equation of motion. Replacing *L* by its value given in Equation (22) resulted the following expression for *C_L_*.

(24) $$C_{L}=\frac{2\;Wn}{\rho_{\infty }SV_{\infty }^{2}},$$

Correspondingly, the drag *D* can be written as the following.

(25) $$D=D(h,V_{\infty },W)=\frac{1}{2}\rho_{\infty }SC_{D0}V_{\infty }^{2}+\frac{2W^{2}n^{2}}{\pi eAR\rho_{\infty }SV_{\infty }^{2}}$$

Thus, Equation (18) becomes the following differential equation for V_∞_.

(26) $$\frac{dV_{\infty }}{dt}=\frac{1}{M}\left\{\;T_{R}-\frac{\left(AFR\right)c}{g}V_{\infty }P-D(h,V_{\infty },W,n)\right\}-g(k\cdot T).$$

The value of *s* is then obtained by solving the following equation.

(27) $$\frac{ds}{dt}=V_{\infty }$$

## 4. Power Setting Optimization Using PSO

**Figure 4:**
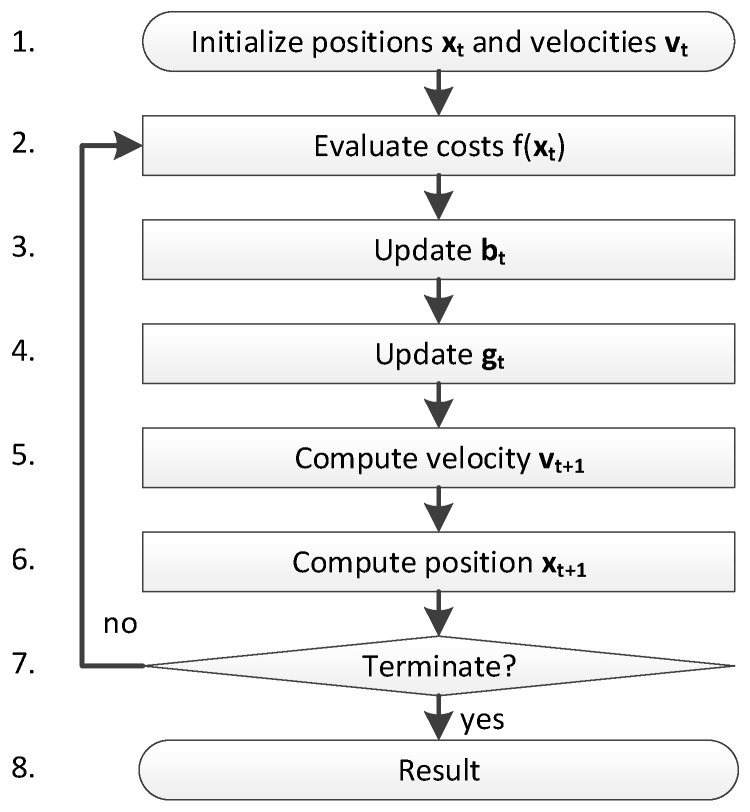
Flowchart of PSO.

PSO is a metaheuristic that was proposed by Kennedy and Eberhart in 1995 [18] and has since been used for finding optimized solutions for a wide range of engineering problems. The algorithm simulates the movements of a swarm of particles in a multidimensional space similar to the movement of a flock of birds or a school of fish. The particles represent the candidate solutions, and their positions evolve throughout the optimization process based on personal and social influences. The flowchart of PSO is illustrated in Figure 4.

In step 1, the particles (i.e., the candidate solutions) and their velocity are randomly initiated over the search space. In step 2, the fitness of each particle is then evaluated using the fitness or evaluation function. For each particle, the best position ${\overset{⃑}{b}}_{t}$ previously occupied by the particle is updated in step 3. This represents the personal influence. Then, in step 4, the best position ${\overset{⃑}{g}}_{t}$ ever occupied by any particle of the swarm is updated. This is the social influence. Based on its personal and the social influence, the velocity and position of each particle are updated in steps 5 and 6 using the equations below.

(28) $${\overset{⃑}{v}}_{t+1}={\omega \overset{⃑}{v}}_{t}+c_{1}{\overset{⃑}{r}}_{1}.\times \left({\overset{⃑}{b}}_{t}-{\overset{⃑}{x}}_{t}\right)+c_{2}{\overset{⃑}{r}}_{2}.\times \left({\overset{⃑}{g}}_{t}-{\overset{⃑}{x}}_{t}\right)$$

(29) $${\overset{⃑}{x}}_{t+1}={\overset{⃑}{x}}_{t}+{\overset{⃑}{v}}_{t+1}$$

where ${\overset{⃑}{r}}_{1}$ and ${\overset{⃑}{r}}_{2}$ are the vectors of random values between 0 and 1, ω is the inertia weight, $c_{1}$ is the personal influence weight and $c_{2}$ is the social influence weight. The termination criterion is checked in step 7. In our case, PSO ran for a predetermined number of iterations before terminating. Finally, the results (i.e., the best global solution ${\overset{⃑}{g}}_{t}$) is returned to the caller.

In our proposed PSO-based algorithm, the candidate solutions represented the power settings along the UAV trajectory. The trajectory was composed of linear segments and circular arcs. However, the output of the smoothing function discussed in Section 2 represented the circular arcs as a sequence of short linear segments. This translated into a trajectory composed only of linear segments in which some were long (segments between the original waypoints) and some were short (segments forming the circular arcs). As a pre-processing step, any segment longer than 500 m was divided into shorter segments. This ensured that the power settings calculated by the proposed algorithm were at a fine resolution. Once the trajectory was divided into a large number of small segments, the segments were processed by PSO in batches of 20. This ensured that the dimension of the problem was not too big and that optimized solutions were found in an acceptable time.

To evaluate the fitness of the candidate solutions, the Runge–Kutta method was used to solve the differential equations presented at the end of Section 3 and compute the speed and weight (i.e., fuel consumption) at the end of each segment starting from the first to the 20th segment of a batch. The constraints on the load factor, the lift coefficient, the maximum speed and the maximum amount of fuel onboard were checked during the calculation. This ensured that the UAV respected its physical constraints.

Since PSO works by improving a population of candidate solutions over a large number of iterations, it can be time consuming to execute. For this reason, we developed a parallel implementation of the algorithm for a multicore CPU. For this implementation, we used OpenMP and multiple threads that evaluated the fitness of the candidate solutions concurrently. This was possible because there were no dependencies between each of the candidate solutions. When running on a multicore CPU, the threads were executed in parallel, accelerating the computation.

## 5. Results

### 5.1. Computing Fuel Consumption at a Constant Power vs. a Constant Speed

**Figure 5:**
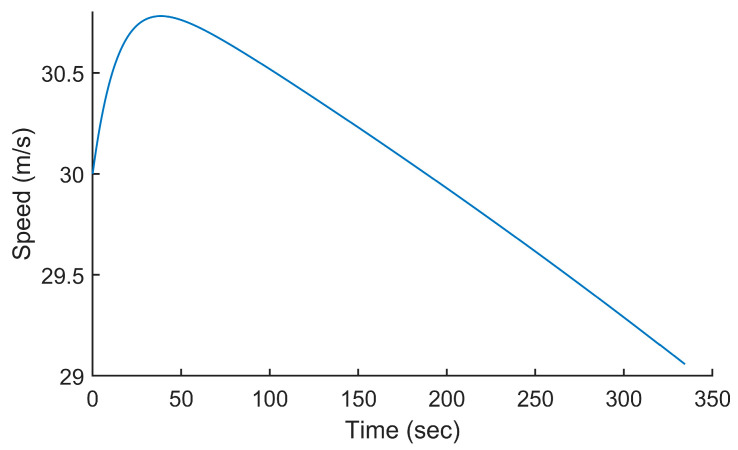
Speed of the UAV along the trajectory P_0_(0, 0, 0) to P_1_(10,000, 0, 1000) when flying at a constant power of 1196 W.

**Figure 6:**
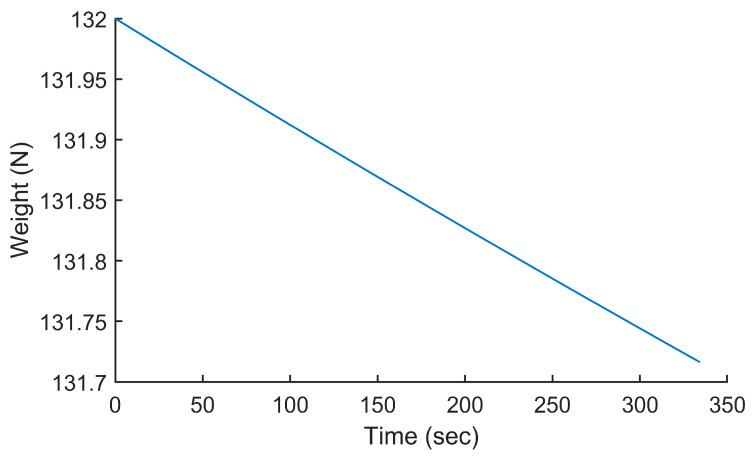
Weight of the UAV along the trajectory P_0_(0, 0, 0) to P_1_(10,000, 0, 1000) when flying at a constant power of 1196 W.

In the first test, we used the analytical model presented in Section 3 to compute the fuel consumption for the Silver Fox UAV, a small 30-pound surveillance UAV whose specifications are given in [19]. This test allowed us to visually confirm the good working of the proposed analytical model and to compare it to the previous works, specifically the one published in [7]. The trajectory used was a linear ascending trajectory starting at P_0_(0, 0, 0) and ending at P_1_(10,000, 0, 1000). The initial altitude was 0 m above mean sea level (AMSL) and the final altitude was 1000 m AMSL. This represented a 10% climb over 10 km. The power setting of the UAV was set to 1196 W so that the average speed of the UAV was 30 m/s along the trajectory. The speed and weight of the UAV are plotted in Figure 5 and Figure 6, respectively. The weight included the fuel and, therefore, its reduction showed the fuel consumption along the trajectory. The total duration of the flight was 334 s. The speed was initially 30 m/s. It increased to 30.78 m/s and reduced to 29.06 m/s at the end of the trajectory. This was because the efficiency of the propeller was higher at a lower altitude and lower at a higher altitude. The initial weight was 132 N and the final weight was 131.716 N, which represented a fuel consumption of 0.284 N. One could intuitively see the non-linearity of the fuel consumption equation; as fuel burned, the UAV got lighter and its fuel consumption got lower.

To compare our study to the previous works, we implemented the method published in [7] and used it to compute the fuel consumption along the same trajectory using a constant speed of 30 m/s. This meant that the power setting of the UAV changed throughout the flight, but this value was not easily observable from the method presented in [7]. The initial weight was 132 N and the final weight was 131.753 N, which represented a fuel consumption of 0.247 N. This represented a difference of 14.9% compared to the fuel consumption calculated by the model presented in this paper. However, although both methods were used for the same trajectory with an average speed of 30 m/s, the method here was used with a constant power settings while the method from [7] was used with a constant speed, which explains the difference in the results.

### 5.2. Experimental Setup

**Figure 7:**
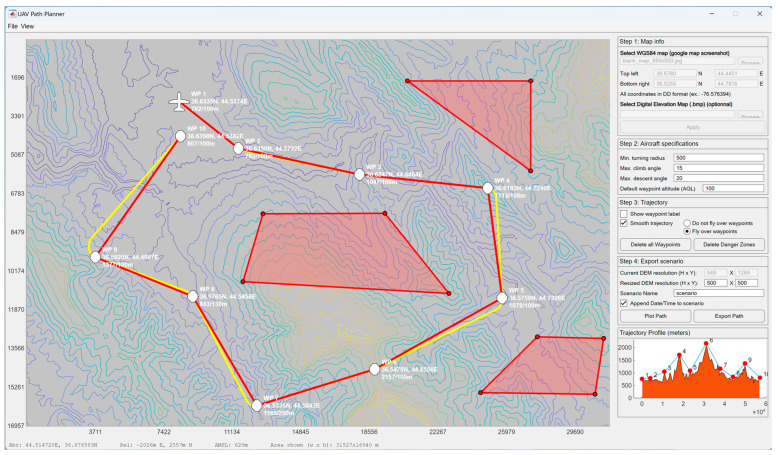
Screenshot of the UAV trajectory planning application showing the waypoints in white, the trajectory in red, the smoothed trajectory that flies over the waypoints in yellow and no-fly zones as red polygons. The elevation profile of the trajectory is shown in the small diagram in the bottom right.

To test the proposed PSO-based algorithm, a graphical user interface (GUI) application, which is shown in Figure 7, was developed. This GUI application was programmed in MATLAB^®^. It allowed for the selection of an area by specifying the coordinates of the top-left and bottom-right corners of the map. Once specified, the application loaded the digital elevation map (DEM) from previously downloaded Shuttle Radar Topography Mission (SRTM) maps. The application then allowed the user to select the initial position of the UAV and to append the waypoints. The elevation of the waypoints could be adjusted using the scroll wheel on the mouse. The red lines represent the segments connecting the waypoints using straight lines. These segments could not be flown as-is by a fixed-wing UAV as they contained discontinuities at the waypoints. The smoothed trajectory, which passes over the waypoints before turning using a circular arc, is shown in yellow. The red polygons represent no-fly zones, which helped the operator plan their mission. On the bottom right of the GUI, one can see the altitude profile of the UAV. This was used to confirm that the trajectory did not collide with the ground.

Once a trajectory was planned in the GUI, the operator clicked on the export button to export the waypoints forming the smoothed trajectory into a text file. This text file was then processed by our proposed PSO-based algorithm to compute the power settings along the trajectory. The PSO-based algorithm was programmed in C++.

### 5.3. Computing the Power Settings to Minimize Fuel Consumption

**Figure 8:**
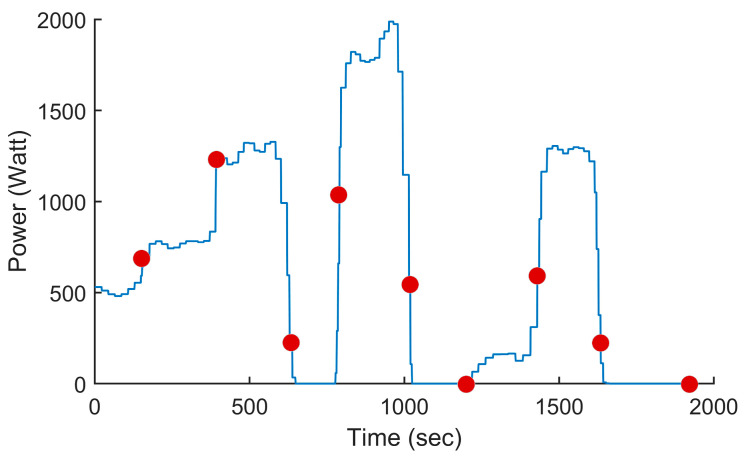
Power setting of the UAV vs. the time for the trajectory shown in Figure 7 when minimizing fuel consumption. The red dots represent the waypoint shown in Figure 7.

**Figure 9:**
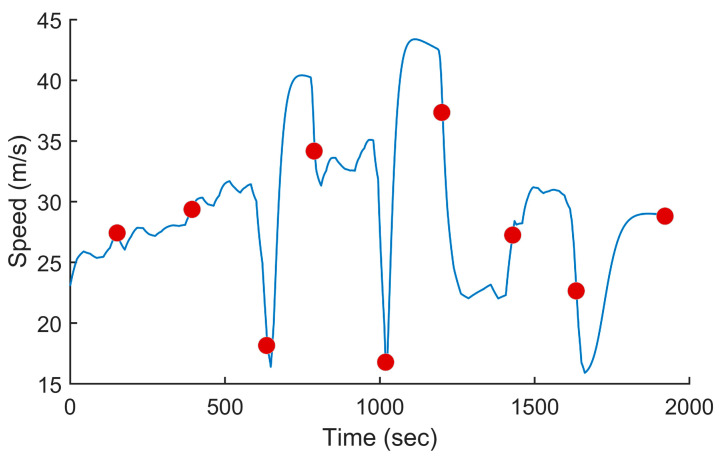
Speed of the UAV vs. the time for the trajectory shown in Figure 7 when minimizing fuel consumption.

**Figure 10:**
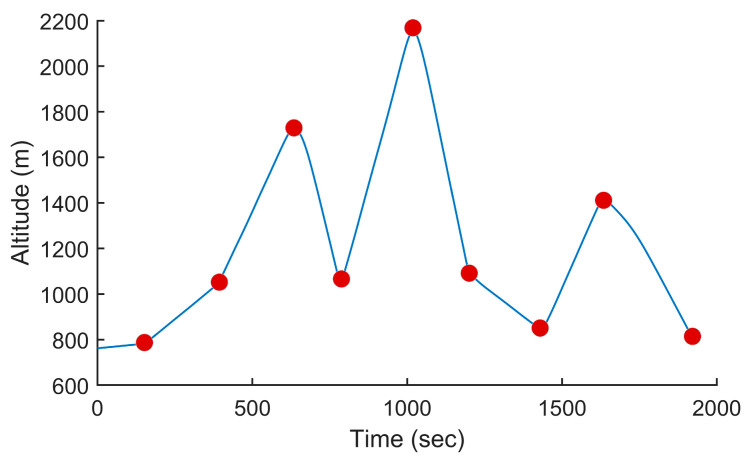
Altitude of the UAV vs. the time for the trajectory shown in Figure 7 when minimizing fuel consumption.

**Figure 11:**
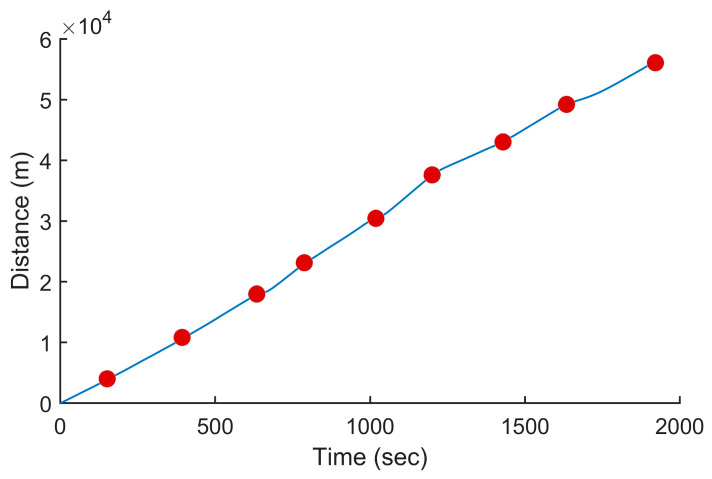
Distance travelled by the UAV vs. the time for the trajectory shown in Figure 7 when minimizing fuel consumption.

**Figure 12:**
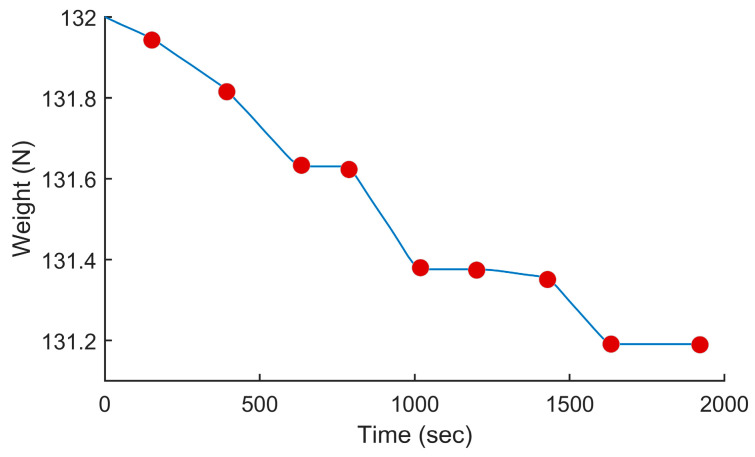
Weight of the UAV vs. the time for the trajectory shown in Figure 7 when minimizing fuel consumption.

In this test, we used the proposed PSO-based algorithm to compute the power settings of the UAV in order to minimize fuel consumption on the overall trajectory. The UAV used in this numerical simulation was still the Silver Fox UAV [19]. The trajectory used was the one shown in Figure 7. It had a length of 56.323 km and was divided into equal segments whose lengths did not exceed 500 m. This ensured a fine granularity when computing the power settings along the trajectory as each small segment received its own power value. This distance could be increased or reduced to accelerate the computation or obtain a higher accuracy as decided by the user. Including the short segments used to form the circular arcs at the waypoints, the segmenting of the trajectory resulted in 125 segments. PSO computed the power settings for the UAV on each segment in order to minimize the fuel consumption while respecting the physical constraints outlined earlier. To reduce the complexity of the problem, the PSO processed the overall trajectory in batches of 20 segments with an overlap of 10 segments between the sections. These dimensions were selected experimentally to ensure a good and fast convergence of PSO. This also meant that PSO required 13 passes to compute the power settings for the 125-segment trajectory used here. PSO was configured with 200 candidate solutions, 1000 iterations, an inertia weight of 0.7298 and a personal and social influence of 1.4960. The power settings, the speed, the altitude of the UAV, the distance travelled by the UAV and the weight of the UAV are shown in Figure 8, Figure 9, Figure 10, Figure 11 and Figure 12, respectively. The decreasing weight of the UAV represented the fuel being consumed by the UAV along the trajectory. In these figures, the red dots represent the waypoints shown in Figure 7. Based on the power settings found by PSO, the UAV could fly the trajectory in 1921.3 s. Its speed varied between 16.49 m/s and 43.33 m/s. Its initial weight was 132.0 N and final weight was 131.192 N, representing a fuel consumption of 0.808 N for a travelled distance of 56.323 km. One can also note that increased power was required when ascending and decreased power was required when descending. In this particular scenario, because the descents were so abrupt, the power settings were set to zero by PSO during the descents, which reduced fuel consumption, but still allowed the UAV to maintain lift.

### 5.4. Computing the Power Settings to Fly at a Constant Speed

**Figure 13:**
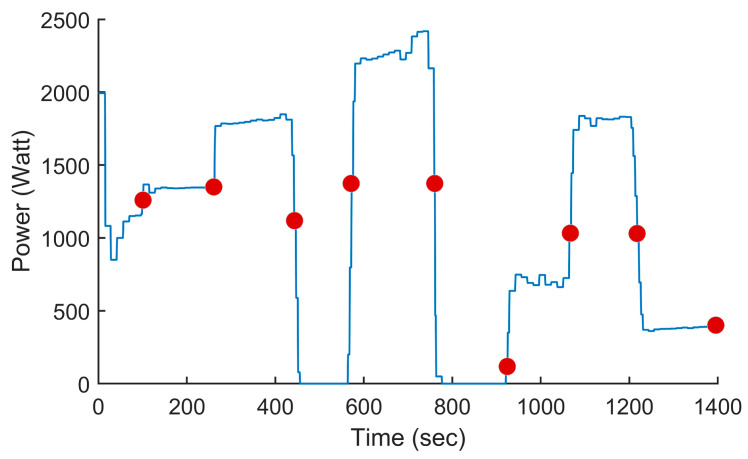
Power setting of the UAV vs. the time when flying at a constant speed.

**Figure 14:**
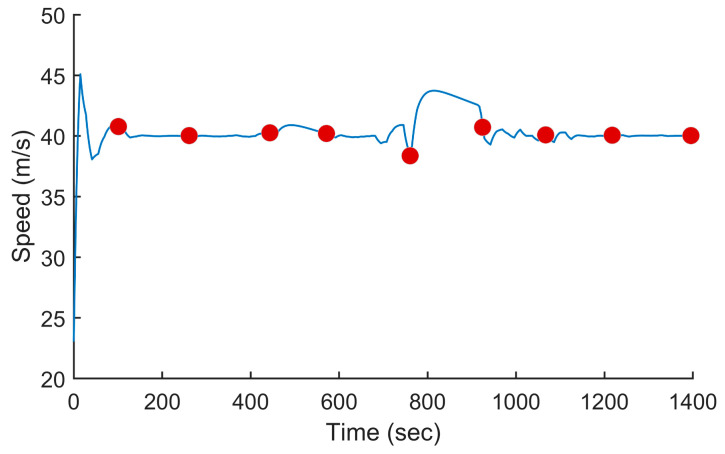
Speed of the UAV vs. the time for the trajectory shown in Figure 7 when flying at a constant speed.

**Figure 15:**
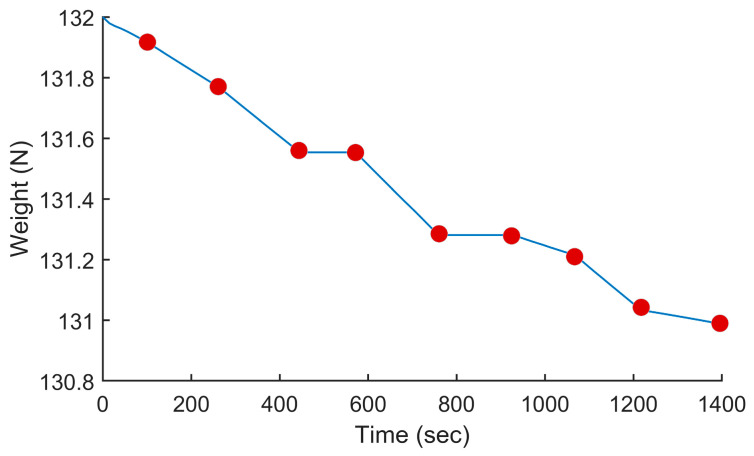
Weight of the UAV vs. the time for the trajectory shown in Figure 7 when flying at a constant speed.

In this test, we modified the objective function of PSO to maintain a constant speed instead of minimizing the fuel consumption. This mimicked the behavior of most UAV autopilots where the operator sets the cruising speed of the UAV at the beginning of the mission and the autopilot maintains that speed throughout the flight. A flight at a constant speed was used here as the baseline to compare our proposed approach, which varied the power settings of the UAV to minimize fuel consumption. In this test, the cruising speed of the UAV was set to 40 m/s. The power settings calculated by PSO and the resulting speed and fuel consumption are shown in Figure 13, Figure 14 and Figure 15. As shown in Figure 14, we can see that the power settings quickly adjusted the initial speed to 40 m/s and ensured a constant speed throughout the trajectory except when the UAV performed a too-rapid descent. When flying at a constant speed, the UAV traveled the trajectory in 1393.8 s. Its initial weight was 132.0 N and its final weight was 130.989 N, which represented a fuel consumption of 1.011 N.

In the previous test, when fuel consumption was minimized, the flying time was longer, but the fuel consumption was 25.51% lower which significantly increased the range of the UAV, confirming the advantage of the proposed PSO-based approach.

### 5.5. Other Scenarios

**Figure 16:**
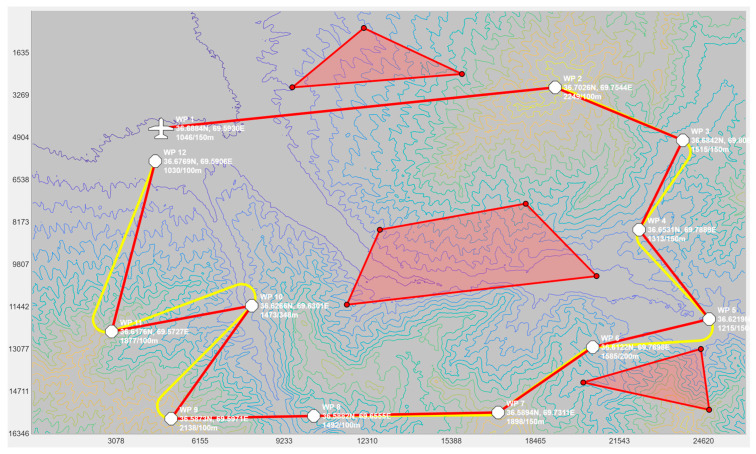
Trajectory for a surveillance mission in Northeast Afghanistan. The waypoints are shown in white, the trajectory in red, the smoothed trajectory that flies over the waypoints in yellow and no-fly zones as red polygons.

**Figure 17:**
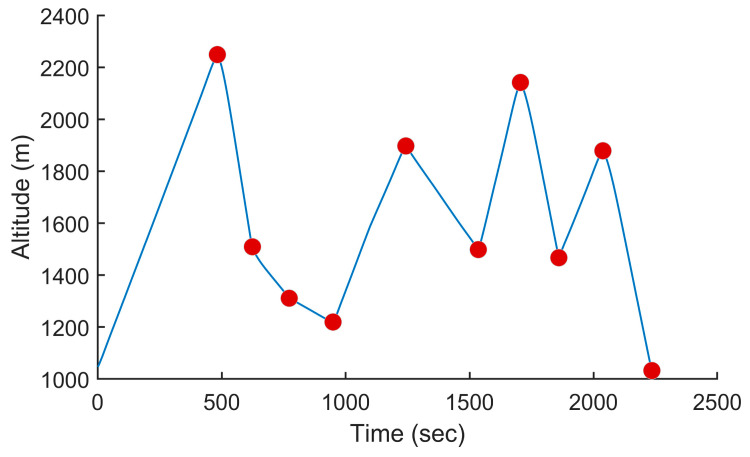
Altitude of the UAV vs. the time for the trajectory shown in Figure 16 when minimizing fuel consumption.

**Figure 18:**
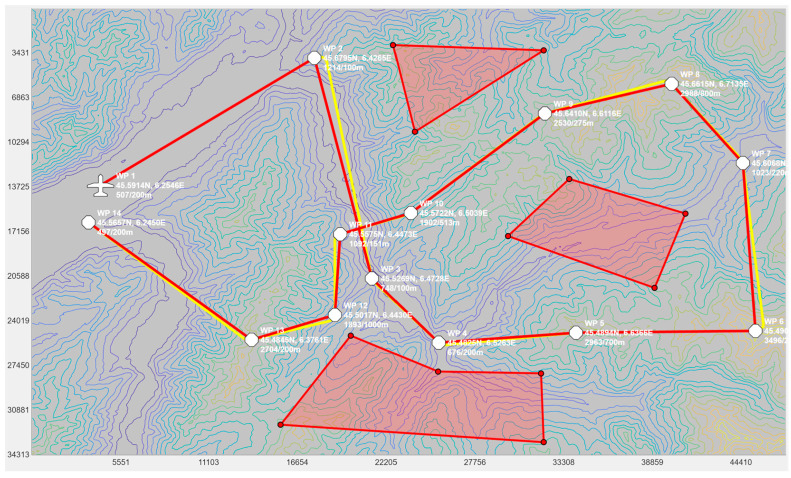
Trajectory for a search and rescue mission in the French Alps. The waypoints are shown in white, the trajectory in red, the smoothed trajectory that flies over the waypoints in yellow and no-fly zones as red polygons.

**Figure 19:**
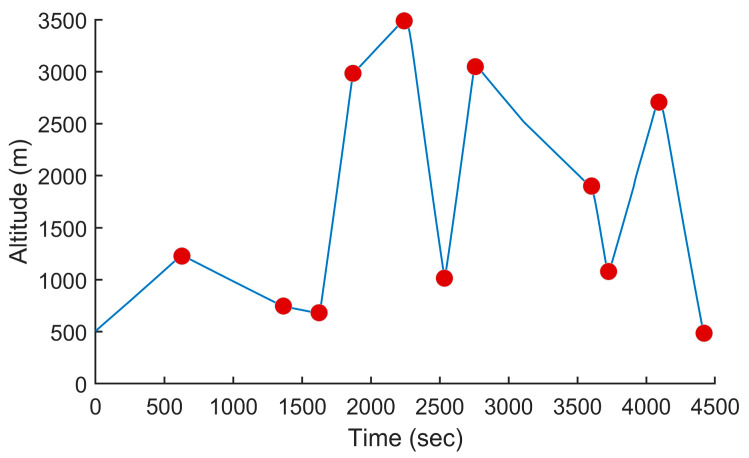
Altitude of the UAV vs. the time for the trajectory shown in Figure 18 when minimizing fuel consumption.

To demonstrate the efficiency of the proposed method on a wider range of trajectories, we performed additional tests using two other trajectories, one from the mountainous regions in Northeast Afghanistan and one in the French Alps, France. The two trajectories are shown in Figure 16 and Figure 18, respectively. The first trajectory could represent a surveillance mission in the context of a military operation, while the second trajectory could be in the context of a search and rescue mission. The altitude profiles of the two trajectories are plotted in Figure 17 and Figure 19. The Afghanistan trajectory had a length of 66.282 km and its altitude varied between 1030 and 2249 m above mean sea level (AMSL). The France trajectory had a length of 133.310 km and its altitude varied between 497 and 3490 m AMSL. The proposed software was used to compute the power settings that minimize fuel consumption for both trajectories. It was also used to compute the power settings to fly at a constant speed. The results for both tests are listed in Table 1. For the Afghanistan trajectory, the fuel consumption was reduced by 23.2% compared to flying at a constant speed. In the case of the France trajectory, the reduction was 20.1%, which was slightly lower, but still an impressive fuel reduction. This showed the efficiency of the proposed algorithm for reducing fuel consumption on various trajectories.

### 5.6. Speedup of the Parallel Implementation

**Figure 20:**
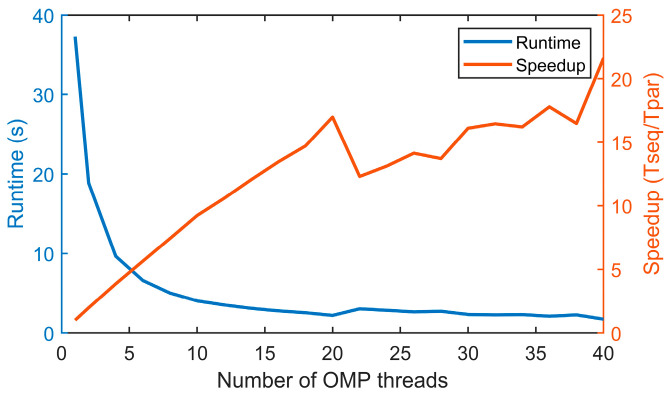
Runtime (s) and speedup (T_sequential_/T_parallel_) of the parallel PSO algorithm implemented in a multicore CPU using OpenMP.

Finally, in the last test, we measured the acceleration brought by the parallelization in the multicore CPU. This test was run on a Dell 7920 workstation equipped with dual Intel Xeon Gold 5218R CPU each with 20 cores for a total of 40 cores running at a base frequency of 2.10 GHz. For this test, we used PSO to optimize the power settings on a 20-segment trajectory and repeated the test multiple times, varying the number of threads used each time. Maximum speedup was expected when the number of threads equalled the number of cores on the computer. The runtime and speedup measurements are illustrated in Figure 20. Using one thread, PSO took 37.27 s to execute and only 1.72 s using 40 threads, which represented a speedup of 21.67×. Based on this runtime, it took 22.36 s for PSO to compute the power settings for the trajectory shown in Figure 7 using 13 passes.

## 6. Discussion

Unlike the previous studies which relied on experimental tables to estimate the fuel consumption of aircraft, this paper presented an accurate mathematical model that computed the fuel burn and velocity of UAVs based on their power settings. This has a great advantage in allowing for the development of optimization algorithms to minimize the fuel consumption and maximize autonomy. In this paper, we used PSO for this purpose and demonstrated using three scenarios that it is possible to minimize fuel consumption by optimizing the power settings of the surveillance UAV while respecting the physical constraints of the UAV, such as the load factor, the lift coefficient, the maximum speed and the maximum amount of fuel onboard. This is one of the first works in the field of fuel consumption minimization for UAVs. Since the method splits the trajectory into shorter fixed-length section and processes them sequentially, it is scalable to longer trajectories, as shown in the results. This is because PSO only needs to deal with the dimension of the short section and not the length of the overall trajectory. Moreover, because the algorithm uses a metaheuristic as its optimization engine, it is possible to extend the fitness function and modify or add additional constraints. The proposed approach also includes path smoothing methods based on circular arcs that overfly the points of interest, which is essential for a surveillance UAV. Finally, the experimental results showed that the proposed method can be efficiently parallelized on multicore processors to accelerate the computation and ensure fast power settings optimization. 

## 7. Conclusions

This paper proposed a PSO-based optimization algorithm to compute the power settings along a flight trajectory in order to minimize UAV fuel consumption. Inputted as a series of waypoints, the trajectory was first smoothed using circular arcs which overflew the waypoints, a desired feature in a surveillance mission. PSO was then used iteratively to try a large number of candidate power settings along the trajectory until it converged to an optimized solution. In the fitness function, an accurate analytical model using the equation of motion of the UAV was used to compute the fuel consumption associated to a given power setting. The absolute physical constraints, such as the load factor, the lift coefficient, the maximum speed and the maximum amount of fuel onboard, were considered in the optimization process. The proposed algorithm was able to reduce the fuel consumption of the UAV by up to 25% in the trajectories used during testing. The algorithm was parallelized in a multicore CPU and achieved a 21.67× speedup compared to a sequential execution in a CPU.

## Figures and Tables

**Table 1:** Results of the proposed method for computing the power settings of the UAV along the trajectory to minimize fuel consumption vs. flying at a constant speed.

Scenario Characteristics			Flying at a Constant Speed		Minimizing Fuel Required		Fuel Required Improvement
Scenario	Location	Length (m)	Flight Duration (s)	Fuel Required (Newton)	Flight Duration (s)	Fuel Required (Newton)	
1	Iraq	56,323	1393.8	1.011	1921.3	0.808	25.1%
2	Afghanistan	66,282	1651.2	1.130	2234.7	0.917	23.2%
3	France	133,310	3228.2	2.402	4413.9	2000	20.1%

## Data Availability

Data are contained within the article.
